# Content validity of the Chinese version of the PRAP scale: a convergent parallel mixed-methods study

**DOI:** 10.3389/frhs.2026.1935953

**Published:** 2026-09-28

**Authors:** Yulin Zhang, Yingwen Wang, Weijia Fu, Beibei Wang, Yanhong Zhang, Qiongfang Kang, Xiaobo Zhang, Ying Gu

**Affiliations:** 1 School of Nursing, Fudan University, Shanghai, China; 2 Nursing Department, Children's Hospital of Fudan University, Shanghai, China

**Keywords:** adolescent, child, mixed method, patient outcome assessment, psychosocial functioning, content validity

## Abstract

**Purpose:**

This study aimed to evaluate the content validity of the Chinese-language version of the PRAP scale from comprehensive perspectives within the COnsensus-based Standards for the selection of health Measurement INstruments (COSMIN) framework.

**Methods:**

We evaluated the content validity of the Chinese PRAP scale using COSMIN methodology in a convergent parallel mixed-methods design (August 2022–November 2023) at a tertiary children's hospital in Shanghai, with 57 content validity raters including nurses, social workers, and family caregivers. Quantitative (I-CVI, S-CVI/Ave) and qualitative data from semi-structured interviews were integrated.

**Results:**

The scale showed strong overall content validity based on the quantitative ratings (S-CVI/Ave = 0.92). Item 5 (“Parent/caregiver stress”) showed the lowest relevance rating (I-CVI = 0.79). Qualitative feedback identified ambiguous phrasing in Items 4 and 5 and inconsistent interpretation of professional terminology.

**Conclusion:**

Despite uncertainties in comprehensibility identified by qualitative insights, the findings provide evidence supporting the content validity of the Chinese PRAP scale, while acknowledging that further psychometric evaluation is required.

## 1 Introduction

Psychosocial risks are defined as social, emotional, familial, and cultural factors that profoundly impact children's health and psychological development (1). Unlike specific clinical conditions such as anxiety and depression, psychosocial risk is a multidimensional construct that reflects a child's overall susceptibility to adverse psychological, emotional, and social outcomes (2). Prevalence studies have demonstrated that 20% to 50% of hospitalized children experience psychosocial risks (2, 3). These risks can lead to serious adverse outcomes, including emotional distress, behavioral disturbances, and impaired social adaptation (4).

Hospitalization exposes children to numerous stressors, including separation from primary caregivers and peers, unfamiliar medical environments, and invasive procedures. These stressors frequently precipitate psychosocial difficulties that may manifest as anxiety and depression. Behavioral responses, such as stress-related disorders and social withdrawal, are also commonly observed (5, 6). These factors can undermine the effectiveness of diagnostic and therapeutic interventions, prolong recovery, and impair children's long-term quality of life (7). Accordingly, early identification of psychosocial risks in hospitalized children is crucial for effective intervention (8). Available screening measures include the Psychosocial Assessment Tool (PAT) (9), the Screen for Child Anxiety Related Emotional Disorders (SCARED) (10), the Children's Depression Inventory (CDI) (11), and the Patient Health Questionnaire for Adolescents (PHQ-9A) (12). However, whereas the PAT directly captures psychosocial risk, the SCARED, CDI, and PHQ-9A assess the clinical manifestations (e.g., anxiety and depression) rather than overall risk. Although these measures are valuable within their respective domains, they present distinct drawbacks for busy clinical settings. The PAT is too time-intensive for routine use, whereas the SCARED, CDI, and PHQ-9A focus primarily on single-symptom domains, limiting their ability to capture multidimensional psychosocial risk.

An efficient screening instrument tailored for healthcare professionals and Child Life Specialists (CLS)—experts in pediatric development and mental wellbeing—is urgently needed. In 2014, Staab et al. developed the Psychosocial Risk Assessment in Pediatrics (PRAP) scale (13), an eight-item measure that enables the rapid identification of high-risk children—with higher scores reflecting greater psychosocial risk—and guides CLSs in designing individualized care plans to enhance the pediatric healthcare experience (14). The PRAP was translated into Chinese using Brislin's back-translation model, demonstrating internal consistency with a Cronbach's *α* of 0.71 (15). Content validity was assessed by an expert panel, and structural validity was evaluated using exploratory factor analysis (15). However, that evaluation was confined to expert ratings of item relevance and did not examine whether key aspects of the construct were missing (comprehensiveness) or whether the items were understood as intended (comprehensibility). As recommended by the COnsensus-based Standards for the selection of health Measurement INstruments (COSMIN) framework, these two dimensions are best evaluated qualitatively through semi-structured interviews with caregivers and healthcare professionals to determine how items are interpreted and identify missing domain content (16). This study aimed to fill this gap by systematically evaluating the content validity of the Chinese version of the PRAP in adherence with COSMIN guidelines.

## 2 Methods

### 2.1 Design

Following COSMIN methodology, a convergent parallel mixed-methods study was conducted from August 2022 to November 2023 at a tertiary pediatric hospital in Shanghai. Quantitative and qualitative data were collected simultaneously, followed by the triangulation of results to comprehensively evaluate content validity. Participants completed structured surveys rating item relevance, comprehensiveness, and comprehensibility, enabling the calculation of the item- and scale-level Content Validity Index (CVI) for the Chinese PRAP scale. To complement the quantitative findings, semi-structured interviews were conducted with stakeholders to gain deeper insight into their perspectives and explain patterns observed in the survey ratings. Reporting adhered to the Good Reporting of A Mixed Methods Study guidelines (17). The research was approved by the Ethics Committee of Children's Hospital of Fudan University (Approval No. 2021378; approved on March 8, 2021).

### 2.2 Participants and eligibility

Participants were recruited from a tertiary children's hospital in Shanghai, China, according to the following inclusion and exclusion criteria:

1. Caregivers
  - Inclusion: Serve as the child's primary caregiver (providing ≥4 h of care daily) (18) and provide informed consent for voluntary participation.
  - Exclusion: The presence of cognitive impairment or mental health conditions (e.g., a current clinical diagnosis of a psychiatric disorder or active psychiatric treatment).
2. Nurses and social workers
  - Inclusion: Hold a valid nursing license or social work certificate, possess at least 3 years of experience in pediatric patient care, and provide informed consent for voluntary participation.
  - Exclusion: Worked fewer than 42 weeks at the hospital in the past year.

### 2.3 Sampling strategy and sample size

For the quantitative phase, convenience sampling was used to recruit family members of pediatric inpatients, nurses, and social workers to form a content validity panel. Given that the COSMIN guidelines consider a representative sample of 50 or more participants sufficient for evaluating measurement properties, a minimum recruitment target of 50 individuals was set (16). The composition of the panel was consistent with current recommendations that representatives of the target population and relevant professionals be involved in content validity evaluation (19).

For the qualitative phase, purposive sampling guided by the principle of maximum variation was adopted (23, 24). A subgroup from the same population was selected to undergo semi-structured interviews concurrently, capturing diversity in patient age, medical condition, and professional perspective. Data saturation was operationally defined as the point at which no new codes or themes emerged across three or more consecutive interviews during ongoing comparative analysis of the transcripts (20).

### 2.4 Content validity evaluation

Content validity was evaluated using complementary quantitative ratings and qualitative interviews designed to address the same core validity questions, with results integrated during data interpretation.

#### 2.4.1 Quantitative approach

Quantitative data on item relevance, comprehensiveness, and comprehensibility were collected from pediatric caregivers and healthcare professionals using rating scales (see Table 1). For the four relevance questions, a 4-point Likert scale was applied: scores of 1 or 2 indicated insufficient relevance to the respective dimension, whereas scores of 3 or 4 indicated good relevance and representativeness (22). The single comprehensiveness question and three comprehensibility questions were scored using a binary method (16, 21). Because the PRAP is a professionally administered rating scale rather than a patient-reported outcome measure, the COSMIN content validity standards were adapted for the evaluator perspective: 8 of the 10 standards were retained, the recall-period standard was omitted as inapplicable to professional administration, and the standards regarding instructions and item comprehensibility were combined into a single question (16, 21).

**Table 1:** COSMIN methodology content validity evaluation items.

Evaluation question		Criteria
Relevance		4-point Likert scale
Question 1	Are the included items relevant for the construct of interest?	
Question 2	Are the included items relevant for the target population of interest?	
Question 3	Are the included items relevant for the context of use of interest?	
Question 4	Are the response options appropriate?	
Comprehensiveness		binary method
Question 5	Are all key concepts included？	
Comprehensibility		binary method
Question 6	Are the PROM instructions understood by the population of interest as intended?	
Question 7	Are the PROM items appropriately worded?	
Question 8	Do the response options match the question?	

COSMIN, consensus-based standards for the selection of health measurement instruments; PROM, proxy-reported outcome measure.

In addition, Question 6 (“whether the items and their response options are understandable”) was adapted from standard COSMIN wording—which assesses target population understanding—to instead evaluate the raters' own understanding. This adjustment reflects that the scale is administered by professionals, making direct evaluator judgment the most relevant indicator of comprehensibility in this context (16). The item-level CVI (I-CVI) for each item–question combination was calculated as the proportion of raters giving a favorable response, defined as “no” regarding missing components for comprehensiveness and “yes” for comprehensibility (19). This proportion-based calculation adhered to standard CVI conventions, wherein an item is deemed content-valid when endorsed by a sufficient proportion of evaluators (19).

#### 2.4.2 Qualitative approach

Qualitative data were gathered through semi-structured interviews with eligible nurses, social workers, and caregivers of hospitalized pediatric patients, employing a descriptive phenomenological approach. The interview guide was developed based on COSMIN content validity evaluation criteria. It focused on the perceived measurement objective of the PRAP scale, negative emotions experienced by children during medical visits, item relevance to the intended construct, target population, and medical environment, item sufficiency in meeting the scale's purpose, and item comprehensibility, including wording appropriateness and option–item alignment.

### 2.5 Data collection

#### 2.5.1 Questionnaire administration

Questionnaires were distributed to caregivers and healthcare professionals on-site and collected immediately upon completion. Each returned questionnaire was reviewed for completeness, and any missing or unclear responses were verified directly with participants for immediate clarification.

#### 2.5.2 Semi-structured interview procedures

Interviews were conducted in quiet, private locations. Before beginning, the study's purpose, methods, and duration were explained, and informed consent for participation and audio recording was obtained. The interviewer guided discussions using a predefined outline, encouraging open dialog while maintaining neutrality and managing session time to keep focus on key research topics.

Following each session, a summary of key points was reviewed with the participant before audio recording ceased. Participants then completed a brief demographic questionnaire assessing gender, age, work experience, professional title, and educational background. Interview durations typically ranged from 25 to 40 min.

### 2.6 Statistical analysis

#### 2.6.1 Quantitative data analysis

Quantitative data were entered into Excel 2010 using double-entry verification to ensure quality control. Continuous data were presented as means ± standard deviation, whereas categorical data were presented as frequencies and percentages. The I-CVI was calculated for each item; an I-CVI ≥0.78 indicated that an item should be retained, a value between 0.70 and 0.77 suggested that revision was required, and a value <0.70 indicated that the item should be excluded (22). The Scale Content Validity Index (S-CVI/Ave) was calculated as the mean of I-CVI values across retained items, with values ≥0.90 signifying good overall content validity (23–25). Item retention criteria and decision timelines were specified *a priori*, before integration with the qualitative strand, to minimize incorporation bias (16).

#### 2.6.2 Qualitative data analysis

Audio recordings from semi-structured interviews were independently transcribed *verbatim* by two postgraduate students and analyzed using content analysis (26, 27). Participant responses were analyzed regarding item relevance, comprehensiveness, and comprehensibility. Relevant qualitative data were subsequently quantified (28, 29), and the degree of endorsement across each PRAP item dimension was statistically analyzed.

#### 2.6.3 Integration of findings

As the qualitative interview guide was directly linked to the quantitative questionnaire items, findings from both strands were integrated using a merged approach, enabling item-by-item and question-by-question comparisons of similarities and discrepancies (30, 31). For each PRAP item and COSMIN question, the quantitative I-CVI values across relevance, comprehensiveness, and comprehensibility (16) were examined alongside corresponding qualitative evidence using a joint display (32). Each item–question combination was then classified into one of three categories: convergence (quantitative and qualitative findings pointed to the same conclusion), discordance (findings conflicted), or complementarity (qualitative data elaborated on or expanded the quantitative results) (33).

## 3 Results

### 3.1 Participant characteristics

A total of 57 valid questionnaires were collected for the quantitative phase. The demographic and professional characteristics of the participants are summarized in Table 2. Healthcare professionals had an average of 10.6 ± 8.2 years of service across specialties, including outpatient and emergency departments, internal medicine, and surgery. The mean age of the children was 5.99 ± 3.79 years, and 79.2% of participating caregivers were female.

**Table 2:** Characteristics of participants.

Items	Category	Subgroups	*n (%)*
Professionals (*n* = 33)	Role	Nurse	29 (87.9%)
		Social worker	4 (12.1%)
	Professional Title	Beginner	13 (39.4%)
		Intermediate	20 (60.6%)
	Education Level	Associate degree or below	6 (18.2%)
		Bachelor degree	19 (57.6%)
		Master degree	8 (24.2%)
Caregivers(*n* = 24)	Family Relationship	Mother	18 (75.0%)
		Father	5 (20.8%)
		Grandmother	1 (4.2%)
	Education Level	Associate degree or below	10 (41.7%)
		Bachelor degree	8 (33.3%)
		Master degree	6(25.0%)

Data are presented as *n* (%). Professional title: “Beginner” and “Intermediate” refer to the entry-level and mid-level professional-title ranks of the hospital, respectively (e.g., junior nurse and nurse-in-charge, respectively).

For the qualitative phase, 18 participants were interviewed, comprising 11 healthcare professionals and 7 caregivers (5 mothers and 2 fathers). Sampling continued until data saturation was achieved, defined as three consecutive interviews yielding no new codes or themes. Participants were assigned codes P1 through P18. The mean interview duration was 28 min per session. Among participating caregivers, educational attainment ranged from primary school to a master's degree, with mothers accounting for 71.4% of caregiver participants.

### 3.2 Validity of the Chinese-language version of PRAP

#### 3.2.1 Item relevance

Quantitative findings: The 57 participants in the quantitative strand rated the PRAP scale's introductory instructions as clear and appropriate. The mean I-CVI for item relevance across all items was 0.89 ± 0.12. Notably, Item 5 (“Parent/caregiver stress”) yielded an I-CVI of 0.79, indicating that it met the predefined threshold of 0.78 for acceptable relevance, although it scored lowest among all items (Table 3).

**Table 3:** I-CVI values for each PRAP item–question combination.

Evaluation question	Item 1	Item 2	Item 3	Item 4	Item 5	Item 6	Item 7	Item 8
Relevance								
Question 1	0.87	0.93	0.90	0.89	0.69	0.90	0.97	0.80
Question 2	0.90	0.93	0.89	0.92	0.77	0.92	0.93	0.82
Question 3	0.73	0.92	0.93	0.93	0.77	0.92	0.90	0.77
Question 4	0.95	0.97	0.97	0.98	0.92	0.95	0.93	0.95
Comprehensiveness								
Question 5	1.00	0.97	0.98	1.00	0.98	0.98	0.98	0.98
Comprehensibility								
Question 6	0.98	0.97	1.00	0.93	0.92	0.87	0.97	0.93
Question 7	0.95	0.93	0.97	0.90	0.95	0.90	0.93	0.93
Question 8	0.98	0.97	0.98	0.93	0.93	0.92	0.98	0.93

I-CVI, item content validity index. Each cell reports the I-CVI value for one item–question combination. The *a priori* item-retention rule was applied at the item level (mean across questions within a dimension); individual question-level values, including those below.70 (e.g., Item 5/Relevance/Question 1 = 0.69), did not lead to item exclusion.

Qualitative findings: During interviews, most PRAP scale items were considered relevant by participants. However, opinions varied regarding Item 4 (“Temperament”) and Item 5 (“Parent/caregiver stress”). Regarding Item 4, participant P2 noted: “Introverted children are not necessarily emotionally stable. Once they reach their anxiety threshold, they are very likely to experience more severe psychological symptoms.” Regarding Item 5, participant P3 questioned: “Can caregiver stress be directly reflected in children? What if the caregiver does not show signs of stress in front of the child?” Overall, qualitative analysis indicated that 71% of interviewees recognized the relevance of Items 4 and 5, while 86% concurred on the relevance of Items 2 and 3.

#### 3.2.2 Item comprehensiveness

Quantitative findings: The I-CVI for item comprehensiveness was 0.98 ± 0.01, indicating that overall comprehensiveness was high based on participant ratings.

Qualitative findings: During interviews, participants frequently proposed additional concepts or domains for potential inclusion. These proposals were categorized into three main themes:

- Concepts implicitly covered: Certain proposed concepts, such as “social needs” and “academic pressure,” were implicitly addressed within existing PRAP items—specifically “Anxiety and Coping in the Medical Environment.” For example, P16 noted, “As soon as I get treated, I want to go back and don't want to stay in the hospital,” and P12 stated, “I hope to get well as soon as possible and then be discharged from the hospital to go to school… I'm a little anxious if I can't be discharged on time.”
- Novel concepts proposed for consideration: Other concepts emerged that are not currently covered in the PRAP scale. For instance, interviewed caregivers introduced the concept of “parental emotional influence,” with P1 asking, “Does the current emotion of family members also have a significant impact on children?” Additionally, participants suggested that integrating comfort objects (such as holding a doll) could serve as a valuable indicator of psychosocial risk, as highlighted by P2: “I've come across some children whose mood seems to be more stable if they have this “comfort object” while hospitalized or undergoing treatment.”
- Insufficient concept explanations: Some participants noted that explanations for certain existing items, particularly “Special needs,” were insufficiently detailed to cover all common conditions in pediatric patients. For example, P11 remarked, “I don't understand why the conditions listed under “special needs” focus entirely on psychological disorders rather than physical health issues.” Overall, all interviewees demonstrated general approval of the scale's comprehensiveness.

#### 3.2.3 Item comprehensibility

Quantitative findings: The I-CVI for comprehensibility was 0.94 ± 0.03, suggesting that overall comprehensibility was high based on quantitative ratings.

Qualitative findings: Participants had varying interpretations of the items, identifying challenges across multiple areas (excluding Item 5):

- Confusion regarding professional terminology: Both caregivers and healthcare professionals expressed confusion regarding specific terms. Caregivers questioned concepts such as “traumatic diagnosis and treatment experience” and “invasive operation/experience.” For instance, P17 asked, “What does this invasive operation mean?”, P2 inquired, “Is there any difference between “traumatic medical experience” and “invasive procedure/experience”?”, and P14 asked, “What does a positive and proactive medical experience mean?” Healthcare professionals, compared to caregivers, exhibited varied interpretations of terms such as “special needs” and “temperament.” P4 stated, “I think children with consciousness disorders also have special needs. This concept explanation does not fully cover the patient population and is prone to variations during assessment. “Special requirements” seem to be the most difficult to determine.” Nurses specializing in child life expressed particular confusion regarding “temperament,” with P8 noting, “I don't quite understand temperament. Is this item evaluating a child's personality traits, or evaluating the child's ability to control emotions?”
- Impact of limited information on assessment accuracy: Some participants noted that limited access to patient information or insufficient familiarity with the child hindered accurate evaluation of anxiety and coping mechanisms. For instance, P1 questioned, “If this is a new patient and I don't have enough information to assess his overall condition, is it possible to make an accurate assessment?”
- Ambiguity of response options: Participants suggested refining the wording of certain response options to improve clarity. For example, it was recommended that the option “Feel relaxed and trust the medical staff” should explicitly specify the context of clinical interactions, with P2 explaining, “Sometimes a child has typical cognitive development and communicates well with parents, but refuses to communicate with healthcare staff.” Additionally, clearer definitions were requested for ambiguous terms such as “certain situations.” P11 noted, “The phrase “in certain situations” in Item 2 is somewhat ambiguous; it is unclear which situations it refers to.”

### 3.3 Synthesis and joint display of the findings

A comparison of quantitative and qualitative findings revealed varying levels of agreement across dimensions. Regarding item relevance, analysis of qualitative interview responses showed that 71% of participants recognized the relevance of Items 4 and 5, whereas 86% endorsed the relevance of Items 2 and 3. For comprehensiveness, participant feedback was universally positive. For comprehensibility, however, the proportion of participants who understood each item varied widely (29% to 71%), with the exception of Item 5, which all participants understood. As shown in Figure 1, this divergence was most pronounced for Items 2, 3, and 4; these items achieved high quantitative CVI values (0.96, 0.98, and 0.92, respectively), even though only 29% of interviewees demonstrated clear comprehension during qualitative interviews.

**Figure 1:**
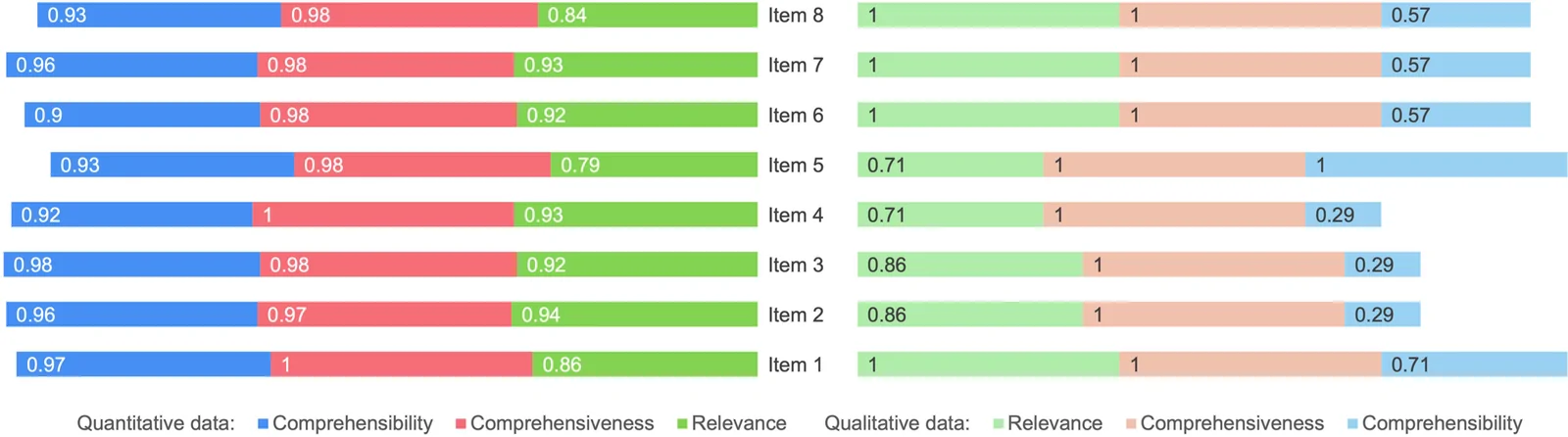
Joint display of quantitative and qualitative results.

Integrated analysis of quantitative and qualitative findings indicated that items with lower content validity (I-CVI below 0.78) in the quantitative strand were predominantly concentrated within the relevance dimension. Conversely, items presenting challenges in the qualitative strand were more frequently observed within the comprehensibility dimension. Each item–question combination was categorized as exhibiting convergence, complementarity, or discordance. These classifications were subsequently integrated with quantitative I-CVI values according to prespecified decision rules, yielding the final recommendation to retain, revise, or exclude each item (Table 4).

**Table 4:** Joint display of quantitative and qualitative findings.

Dimension	Code	Quantitative findings	Qualitative findings	Results of integration	Revision decision
Relevance					
Item 1	Q4	I-CVI = 0.89	“I feel that temperament is more related to the child's personality or individual traits.”	Complementarity	
	Q5	I-CVI = 1.00	“It's not about correlation, because you are assessing the child.”	Convergence	Retained with clarification.
Item 2	Q2	I-CVI = 0.93	“The listed diseases don't seem to fully cover our patient population.”	Discordance	
	Q3	I-CVI = 0.89	“I want to know if the assessor conducts on-site evaluations or makes judgments based on previous information obtained from parents.”	Complementarity	
	Q5	I-CVI = 0.97	“The assessment is for the child… why consider parental stress?”	Complementarity	Needs to be revised.
Item 3	Q2	I-CVI = 0.92	“The “special needs” Qis not very applicable to our ward.”	Discordance	
	Q3	I-CVI = 0.93	“If a child has practically no medical history, how can we determine if they will trust medical staff in the future?” “This item's subjective nature might lead to significant scoring discrepancies.”	Complementarity	
	Q4	I-CVI = 0.93	““Limited or unpredictable adaptability” seems difficult to define.”	Discordance	
	Q5	I-CVI = 0.98	“Can it absolutely reflect the child's (situation)? Or only partially?”	Complementarity	Needs to be revised.
	Q8	I-CVI = 0.98	“When assessing this item, will the influence of anxiety levels be mixed in?”	Convergence	Needs to be revised.
Item 4	Q3	I-CVI = 0.97	“How do we assess whether the child has moderate anxiety?”	Complementarity	
	Q4	I-CVI = 0.98	“It feels like it doesn't quite match the options.”	Discordance	
Comprehensiveness					
Item 5	Q1∼8	I-CVI = 0.97～1.00	/	Convergence	
Comprehensibility					
Item 6	Q1	I-CVI = 0.98	““Limited communication ability” – limited by what?”	Complementarity	
	Q2	I-CVI = 0.97	“I don't quite understand this item.”	Discordance	
	Q3	I-CVI=1.00	“I'm not sure which situations it refers to.”	Complementarity	
	Q4	I-CVI = 0.93	“Is this for evaluating a child's underlying personality traits? Or is it for evaluating a child's emotional control ability?”	Discordance	
	Q6	I-CVI = 0.87	“What degree of impact marks the threshold for progressing to the next stage?”	Complementarity	
	Q7	I-CVI = 0.97	“What constitutes “moderate impact”?”	Complementarity	
Item 7	Q1	I-CVI = 0.95	“The phrase “in communication” could be added to the options.”	Complementarity	
	Q2	I-CVI = 0.93	“How do we differentiate between the concepts of “frequently” and “continuously”?”	Complementarity	
	Q3	I-CVI = 0.97	““Rarely or no anxiety” is somewhat easier to understand… What circumstances qualify as moderate anxiety?”	Complementarity	
	Q4	I-CVI = 0.90	“Why not use “adaptability” to summarize this item?”	Discordance	
	Q6	I-CVI = 0.90	“If it includes unpleasant experiences, is this considered relatively positive or negative? The concept of “relative” could be defined more clearly.”	Complementarity	
	Q7	I-CVI = 0.93	“It's hard to distinguish between mild, moderate, and severe.”	Discordance	
	Q8	I-CVI = 0.93	“It's still based on subjective feeling, there isn't a specific standardized boundary.”	Discordance	
Item 8	Q2	I-CVI = 0.97	“The concept itself is ambiguous, so the evaluation is likely to be even more ambiguous.”	Discordance	
	Q4	I-CVI = 0.93	“I would feel that (this item) refers to (the child's) adaptability to the environment.”	Discordance	

Only item–question combinations with qualitative concerns or borderline I-CVI values are shown. I-CVI, content validity index. Item 1: Communication, Item 2: Special needs, Item 3: Anxiety and coping in the clinical setting, Item 4: Temperament, Item 5: Parental/caregiver stress, Item 6: Past medical experiences, Item 7: Invasive procedures/experiences, Item 8: Impact of developmental stage on coping ability.

## 4 Discussion

This study evaluated the content validity of the PRAP scale using a mixed-methods design. The scale achieved an S-CVI/Ave of 0.92, indicating strong overall content validity based on the quantitative ratings. However, a high CVI demonstrates only that items are perceived as relevant, comprehensive, and clear; it does not independently establish overall measurement validity or prove that the scale accurately represents its underlying construct. Structural validity—assessed, for instance, through exploratory factor analysis using principal component analysis with varimax rotation—is required to support such claims (16) and is addressed in the Chinese adaptation and validation study of the PRAP scale (15).

Nevertheless, the high aggregate index did not mask item-level weaknesses. Several items exhibited lower I-CVI values, most notably Item 5, whose relevance I-CVI was 0.79 (just above the 0.78 retention threshold). Furthermore, participant comprehension during qualitative interviews varied widely across items (29%–71%). These discrepancies were identified through the joint display (Table 4 and Figure 1) and resolved through targeted item revisions, which focused on clarifying item wording rather than deleting items. Although the *a priori* decision rules included an exclusion threshold, no item-level I-CVI fell below it, and all eight items were retained. This underscores the necessity of a mixed-methods approach to evaluate content validity beyond aggregate indices alone.

### 4.1 Analysis of the PRAP scale relevance

Among the items evaluated for relevance, “Temperament” (Item 4) and “Parent/caregiver stress” (Item 5) raised the most concern across both strands. Quantitatively, Item 5 yielded the lowest relevance I-CVI (0.79), while qualitative feedback reflected uncertainty regarding how broadly both constructs should apply. These concerns align with existing literature on both constructs. Regarding temperament, research has shown that this item has complex associations with behavioral problems, emotion regulation, and mental health (34). Children with low activity levels and limited self-regulation are prone to externalizing problems, such as attention-deficit/hyperactivity disorder (35), traits that can prompt impulsive behavior and poor adaptation when facing environmental challenges. For caregiver stress, contributing factors extend well beyond financial strains to include unfamiliarity with medical procedures and anxiety arising from information asymmetry with medical staff (36). Caregiver stress often stems from unmet medical and emotional needs, and the caregiver's emotional state, behavior, and coping patterns are subtly transmitted to the child.

### 4.2 Analysis of the PRAP scale comprehensiveness

The comprehensiveness of the PRAP scale received high quantitative ratings (mean I-CVI = 0.98), indicating that no key domain of psychosocial risk was considered missing. Qualitative interviews nevertheless highlighted additional concepts relevant to psychosocial risk. Higher parental education levels frequently correlate with supportive parenting practices that foster cognitive and emotional development, thereby mitigating psychosocial risk (37). Similarly, caregiver–child attachment quality contributes directly to socioemotional development (38). Finally, reliance on a comfort object can support self-soothing during acute stress (39), though this strategy is largely confined to early childhood (approximately 6–24 months) and does not reflect a child's broader emotion-regulation capacity.

Because these concepts fell outside the predefined construct, they were not added to the PRAP scale in the present study. Instead, they offer hypotheses for future scale expansion, which would require formal expert panel appraisal and psychometric evaluation. Consequently, the existing item set was retained as comprehensive, with these additional constructs noted as avenues for future instrument development.

### 4.3 Analysis of the PRAP scale comprehensibility

Regarding comprehensibility, it is essential to distinguish between “traumatic experience” and “invasive procedure/experience” for accurate scale administration. A traumatic experience reflects the child's subjective perception of people, events, and objects encountered during medical treatment, encompassing their perspective on diagnostic and treatment outcomes regardless of whether those outcomes are positive or negative. An invasive procedure/experience, by contrast, evaluates the observable impact of medical procedures on the child from an objective, clinical standpoint. This distinction supports the use of broader phrasing such as “specific situations” and “adaptability may vary” within item wording. To administer the PRAP scale effectively, assessors must establish a clear, shared understanding of what each item denotes prior to assessment (40). These items achieved high quantitative comprehensibility I-CVI values, corroborating qualitative findings that their wording remains appropriate once these conceptual differences are clarified.

### 4.4 Quantitative–qualitative divergence informs item revisions

The quantitative and qualitative findings revealed a complementary divergence: item–question combinations with an I-CVI below 0.78 fell within the relevance dimension, whereas items generating the strongest conceptual debate in qualitative data were located within the comprehensibility dimension. This asymmetry indicates that perceived weaknesses in relevance often stemmed from misunderstandings of item wording or response options. Regarding the “special needs” item, the disease examples listed in the scale explanation failed to encompass all children with special health needs (41). This limitation rendered the item difficult to apply across diverse medical scenarios, which reduced its perceived relevance and introduced ambiguity into raters' judgments (41). A broader definition of “special needs” was therefore adopted—encompassing children with chronic physical, developmental, or mental conditions requiring health services beyond daily care (39)—while recognizing that children with conditions such as autism spectrum disorder present distinct sensory needs (40). Regarding the “temperament” item, interview participants found it particularly difficult to understand. Interpreting the term narrowly as “character” or “emotion stabilization” was deemed insufficient, creating the perception that temperament was only weakly related to psychosocial risk and thereby lowering its relevance score (37). Consequently, “temperament” was reframed to focus on the child's emotional state in the immediate medical situation, rather than relying on past or trait-based behaviors.

### 4.5 Limitations

Several limitations warrant consideration. First, single-center convenience sampling in Shanghai limits the generalizability of the findings, highlighting the need for multicenter validation. Second, the predominance of female caregivers may underrepresent male perspectives, though this likely reflects prevailing caregiving demographics. Third, eligibility criteria—requiring at least 42 weeks of hospital employment within the past year for expert participants and excluding caregivers with diagnosed mental health disorders—may introduce selection bias and reduce sample representativeness. Fourth, qualitative interviews were conducted by investigators familiar with the PRAP construct, meaning researcher preconceptions cannot be entirely excluded, nor can the full spectrum of external perspectives be guaranteed. Despite these limitations, this study provides the first COSMIN-guided, mixed-methods evaluation of the content validity of the Chinese version of the PRAP scale, offering a defensible foundation for its clinical use and future multicenter validation.

## 5 Conclusions

Grounded in COSMIN methodology, this study integrated feedback from pediatric patient families and clinical experts to evaluate the Chinese version of the PRAP scale. The findings provide evidence supporting the content validity of the Chinese version of the PRAP scale for assessing pediatric psychosocial risk, while acknowledging that further psychometric evaluation is required.

## Data Availability

The raw data supporting the conclusions of this article will be made available by the authors, without undue reservation.
